# Smooth semi‐nonparametric (SNP) estimation of the cumulative incidence function

**DOI:** 10.1002/sim.7331

**Published:** 2017-05-23

**Authors:** Anh Nguyen Duc, Marcel Wolbers

**Affiliations:** ^1^ Oxford University Clinical Research Unit Wellcome Trust Major Overseas Programme Ho Chi Minh City Vietnam; ^2^ Centre for Tropical Medicine, Nuffield Department of Medicine University of Oxford Oxford U.K.

**Keywords:** competing risks, cumulative incidence function, smooth semi‐nonparametric (SNP) estimation, mixture factorization, interval censoring

## Abstract

This paper presents a novel approach to estimation of the cumulative incidence function in the presence of competing risks. The underlying statistical model is specified via a mixture factorization of the joint distribution of the event type and the time to the event. The time to event distributions conditional on the event type are modeled using smooth semi‐nonparametric densities. One strength of this approach is that it can handle arbitrary censoring and truncation while relying on mild parametric assumptions. A stepwise forward algorithm for model estimation and adaptive selection of smooth semi‐nonparametric polynomial degrees is presented, implemented in the statistical software R, evaluated in a sequence of simulation studies, and applied to data from a clinical trial in cryptococcal meningitis. The simulations demonstrate that the proposed method frequently outperforms both parametric and nonparametric alternatives. They also support the use of ‘ad hoc’ asymptotic inference to derive confidence intervals. An extension to regression modeling is also presented, and its potential and challenges are discussed. © 2017 The Authors. *Statistics in Medicine* Published by John Wiley & Sons Ltd.

## 1 Introduction

Competing risks refer to the situation where multiple event types are of interest and the time *T* and the event type *D* of the first occurring event, possibly subject to censoring or truncation, is analyzed. Competing risks are frequent in medical research, especially in multimorbid or critically ill study populations 1. The most important descriptive quantity in the analysis of competing risks data is the cumulative incidence function (CIF), which measures the absolute risk of different event types *j* over time: *C* *I* *F*_*j*_(*t*)=*P*(*T*⩽*t*,*D*=*j*). In the presence of right censoring or left truncation, the CIF is usually estimated by the nonparametric Aalen–Johansen estimator, which gives a step‐function 2. Non‐parametric estimates of the CIF for interval‐censored data have also been suggested, but they are non‐unique over certain sub‐intervals of the follow‐up period and may have nonstandard asymptotic behavior with convergence rates slower than *n* ^−1/2^ 3, 4, 5. Parametric methods for CIF estimation provide smooth estimates, can easily be applied to arbitrary censoring and truncation schemes, have standard asymptotic properties, and may be more efficient if the assumed parametric model is correct 6, 7. However, they rely on parametric assumptions, which may not be tenable.

In econometrics and survival analysis, smooth ‘semi‐nonparametric’ (SNP) densities have been successfully used as building blocks for flexible models that combine some of the advantages of nonparametric and parametric approaches 8, 9, 10. In this work, we propose a novel SNP estimator of the CIF. The rest of this paper is organized as follows: Section 2 gives a brief overview of SNP density estimation. Section 3 describes the underlying model for the CIF, and Section 4 proposes an estimation algorithm and methods for ‘ad hoc’ asymptotic inference. A test statistic to compare the CIFs for a specific event type between independent groups is given in Section 5. Section 6 evaluates the finite‐sample performance of the proposed methods in a simulation study, and Section 7 gives an application to a trial in cryptoccocal meningitis. The potential and challenges of extending our CIF‐estimation approach to regression modeling are discussed in Section 8, followed by concluding remarks.

## 2 Smooth semi‐nonparametric density estimation – overview

The SNP densities were first introduced in the field of econometrics by Gallant and Nychka 8 to estimate the distribution of a nuisance random part in a statistical model where little is known about the shape of the distribution. For univariate problems, an SNP density is of the form $g_{K}(z)=P_{K}^{2}(z)\psi (z)+\epsilon_{0}g_{0}(z)$ where $P_{K}(z)=\sum_{i=0}^{K}a_{i}z^{i},\psi (z)$ is a known base density such as the standard normal density, and *g*_0_(*z*) is a known strictly positive density with expectation zero, which together with the small positive constant *ϵ*_0_ governs the tail behavior of *g*_*K*_(*z*). For fixed *K*,*g*_*K*_(*z*) is fully parametrized by the finite set of polynomial coefficients $\left(a_{0},\ldots ,a_{K}\right)$, which can be estimated using standard techniques such as maximum‐likelihood estimation (MLE). If the polynomial degree *K*=*K*_*n*_ is adaptively chosen depending on the sample size *n* such that $\lim_{n\to \infty }K_{n}=\infty$, then $g_{K_{n}}(z)$ can consistently estimate any density belonging to a rich class of smooth densities allowing for skewness, kurtosis, multi‐modality, or non‐normal tail behavior under certain regularity conditions 8. Moreover, it was demonstrated in an extensive simulation study that SNP density estimation is qualitatively similar to kernel density estimation 11.

Smooth semi‐nonparametric densities were introduced as building blocks for regression modeling of time‐to‐event data and estimation of the survival function by Zhang, Doehler, and Davidian 9, 10. For estimation of the survival function of a random variable *T*, a simple model of the form

(1) $$logT=\mu +\sigma Z,\mu \in R,\sigma \in R^{+}$$

is used with *μ* and *σ* governing the location and scale of logT, respectively, and *Z* is assumed to have a SNP density of the form $h_{K}(z)=P_{K}^{2}(z)\varphi (z)$, where *φ*(*z*) is the density function of the standard normal distribution and the ‘tail’ *ϵ*_0_ *g*_0_(*z*) is ignored as in most practical implementations of the methodology. Alternatively, the authors suggested to parametrize the distribution of *e* ^*Z*^ by a SNP density of the form $l_{K}(z)=P_{K}^{2}(z)\varepsilon (z)$ where *ε*(*z*) is an exponential density with rate 1. For fixed *K*, the distribution of *T* in (1) only depends on *μ*,*σ* and the polynomial coefficients of the SNP density, leading to a tractable implementation of the likelihood function under arbitrary censoring and truncation. The choice of *K* as well as the choice between a normal or exponential base density is made adaptively based on an information criterion such as the criterion proposed by Akaike. In extensive simulation studies, several authors 9, 10 demonstrated the advantages of the SNP approach compared with traditional parametric, semi‐parametric and nonparametric methods. Moreover, they ascertained earlier claims (e.g. 11) that ‘ad hoc’ asymptotic inference that ignores the adaptive choice of *K* frequently provides confidence intervals with acceptable coverage.

## 3 A mixture model for the cumulative incidence function based on smooth semi‐nonparametric densities

We assume that competing risks data, that is, the time to the first event *T* and the respective event type *D*, possibly subject to censoring and truncation, are available for a sample of *n* independent subjects. The event type *D* can take any value in $\left\{1,\ldots ,J\right\}$, where *J* denotes the total number of event types. The CIF for event type *j* is defined as *C* *I* *F*_*j*_(*t*)=*P*(*T*⩽*t*,*D*=*j*). Our model relies on a mixture factorization that factorizes the CIF into a product of the marginal probability of the event type *j* and the probability of surviving up to time *t* conditional on eventually experiencing an event of type *j*:

(2) P(T⩽t,D=j)=P(T⩽t|D=j)P(D=j)

This factorization has been used by several authors 12, 13, 14. However, it has also been criticized because the marginal event probabilities, *P*(*D*=*j*), are poorly identified for data with a limited follow‐up duration relative to the timing of events, and because of interpretational issues due to conditioning on the future event type *D* 15. Here, we only use this factorization as a convenient mathematical tool for model formulation and focus our attention on CIF estimation over the observed follow‐up period. We will revisit whether the identifiability issue affects our CIF estimates in an extensive simulation study (Section 6) and the discussion.

The marginal event probabilities *P*(*D*=*j*) are assumed to follow a simple multinomial model that we parametrize by a multinomial logistic model with intercept terms only:

(3) $$P\left(D=j\right)=\exp\left(\gamma_{j}\right){\left\{1+\overset{J-1}{\underset{k=1}{\sum }}\exp\left(\gamma_{k}\right)\right\}}^{-1}$$

where *γ*_*j*_,*j*=1,…,*J*−1, are the parameters, and *γ*_*J*_ is set to 0 to ensure model uniqueness.

Conditional on *D*=*j*,*T*|*D*=*j* is a ‘proper’ time‐to‐event variable, and we parametrize its distribution using SNP densities as suggested in 9, that is, we assume that for j=1,…,J

(4) $$log\left(T|D=j\right)=\mu_{j}+\sigma_{j}Z_{j},\;\mu_{j}\in R,\sigma_{j}\in R^{+}$$

where *Z*_*j*_ are random variables whose distribution is modeled using SNP densities, and *μ*_*j*_ and *σ*_*j*_ govern the location and scale of $log\left(T|D=j\right)$, respectively. As in 9, we consider two different SNP models for *Z*_*j*_: (1) a direct model that assumes that *Z*_*j*_ follows a SNP density of degree *K*_*j*_ with a standard normal base density, or (2) an indirect model that models $e^{Z_{j}}$ based on a SNP density with an exponential base density with rate 1. For parsimony reasons, we assume that all conditional time‐to‐event distributions use the same base density but possibly different polynomial degrees *K*_*j*_ and parametrize the polynomial coefficients of the SNP densities by spherical coordinates $\phi_{K_{j}}=\left\{\phi_{jk}\in (-\pi /2,\pi /2];\;k=1,\ldots ,K_{j}\right\}$ as detailed in the web‐appendix of 9. Of note, for the special case of *K*_*j*_=0, the model specifies a log‐normal or a Weibull distribution for the conditional time‐to‐event distributions.

## 4 Model estimation and statistical inference

### 4.1 Likelihood construction for fixed polynomial degrees

Direct simultaneous estimation of all parameters in the model specified previously including the SNP degrees $K=\left\{K_{1},\ldots ,K_{J}\right\}$ is not feasible. However, for fixed base densities and a fixed set of SNP polynomial degrees **K**, the underlying model is parametric with a total parameter set given by $\theta (K)=\left(\cup_{j=1}^{J}\left\{\mu_{j},\sigma_{j},\phi_{K_{j}}\right\}\right)\cup \left(\cup_{j=1}^{J-1}\left\{\gamma_{j}\right\}\right)$. Hence, standard MLE of *θ*(**K**) can be employed. Importantly, the likelihood can easily be specified for arbitrary censoring and truncation mechanisms. For example, for competing risks data subject to independent right or interval censoring as well as left‐truncation, the respective likelihood contribution from a subject is

(5) $$\frac{P{\left(T>t_{l}\right)}^{I\left(D^{\prime }=0\right)}}{P\left(T⩾l\right)}\times \overset{J}{\underset{j=1}{\prod }}{\left[\;f_{j}{\left(t_{l}\right)}^{I\left(t_{r}=t_{l}\right)}{\left\{P_{j}\left(T⩽t_{r}\right)-P_{j}\left(T⩽t_{l}\right)\right\}}^{I\left(t_{r}>t_{l}\right)}\right]}^{I\left(D^{\prime }=j\right)}$$

for 0⩽*l*<*t*_*l*_⩽*t*_*r*_. Here, $D^{\prime }$ refers to the observed event type indicator with $D^{\prime }=D$ if an event was observed at time *t*_*l*_=*t*_*r*_ (for uncensored data) or in the interval $\left[t_{l},t_{r}\right]$ (for interval‐censored data), and $D^{\prime }=0$ refers to a right‐censored observation at time *t*_*l*_. *l* refers to the truncation time, *f*_*j*_(*t*)=*f*_*T*|*D*=*j*_(*t*)*P*(*D*=*j*), where *f*_*T*|*D*=*j*_(*t*) is the density of *T*|*D*=*j*, and *P*_*j*_(*T*⩽*t*)=*P*(*T*⩽*t*|*D*=*j*)*P*(*D*=*j*). *P*(*T*⩽*t*) equals $1-\sum_{i=1}^{J}P_{j}(T⩽t)$. Explicit formulas for *P*(*T*⩽*t*|*D*=*j*) can be derived using integration by parts as described in 9.

### 4.2 Estimation procedure

For a fixed base density, we propose to choose the polynomial degrees based on an information criterion using the following greedy step‐wise forward algorithm:

Step 0: Perform MLE for the parametric model with all *K*_*j*_=0. This is the current best fit. Go to Step 1.
Step 1: Calculate MLE for the *J* parametric models that correspond to increasing the polynomial degrees *K*_1_,…,*K*_*J*_, one at a time, by 1 compared with the current best fit. Among these, we call the model with the best information criterion the new best model.
Step 2: If the new best model is better than the current best fit, replace the current best fit with the new best model and repeat Step 1. Otherwise, go to Step 3.
Step 3: Stop and return the current best fit.

In practice, we truncate the maximum allowed polynomial degrees at $K_{\max}$ for computational reasons. In survival analysis, $K_{\max}=2$ is generally sufficient to achieve an excellent fit based on extensive simulation studies 9; in other settings, values of $K_{\max}$ from 2 to 4 have been suggested 11. Commonly used information criteria are of the form $2\times \left\{-l(\theta (K))+qc\right\}$, where *l*(*θ*(**K**)) is the log‐likelihood evaluated at the MLE for fixed polynomial degrees and *q* is the total number of parameters in the model. Typical choices of *c* are *c*=1 (Akaike's information criterion, AIC), c=log(n)/2 (Bayesian information criterion, BIC*n*), and c=log{log(n)} (Hannan–Quinn's criterion, HQC*n*). Alternatively, we also considered replacing *n*, the sample size, by *d*, the total number of events of any type, in the definition of BIC and HQC and denote the resulting information criteria by BIC*d* and HQC*d*, respectively. A schematic of the greedy stepwise forward algorithm for 2 competing risks with $K_{\max}=2$ and AIC as the information criterion is given in Appendix A of the Supporting Information. Finally, the choice between the normal and the exponential base density is made by comparing the best fits from both base densities using the same information criterion.

For fixed polynomial degrees, the log‐likelihood function is still relatively complex and may have multiple extrema. Hence, good starting values for the numerical optimization algorithm are crucial. We detail the proposed selection of starting values in Appendix B of the Supporting Information. In brief, for step 0, starting values for *μ*_*j*_ and *σ*_*j*_ are determined based on parametric log‐normal or Weibull survival models including all subjects with an observed (or interval‐censored) event of type *j*. Right‐censored observations are also included but weighted based on a crude estimate of their probability of ultimately experiencing event type *j*. Initial values for *γ*_*j*_(*j*=1,…,*J*−1) are then obtained by optimizing the likelihood function with respect to these parameters only. For step 1, starting values for *γ*_*j*_ are the estimates from the current best fit. For the new polynomial coefficients (in spherical coordinates), multiple starting values are selected from a regular grid on ${\left[-\pi /2,\pi /2\right]}^{K_{j}+1}$ as suggested in 9. Finally, corresponding new starting values for *μ*_*j*_ and *σ*_*j*_ are selected such that the first two moments of the distribution of logT|D=j (which depend on these parameters and the spherical coordinates) remain unchanged compared with the current best fit. For each set of starting values, numerical optimization of the log‐likelihood is then implemented using a quasi‐Newton algorithm.

An implementation of the proposed estimation algorithm by the first author using the statistical software R 16 has been uploaded to GitHub (see Section 9 for details).

### 4.3 Ad hoc statistical inference for cumulative incidence function estimates

A rigorous proof of asymptotic normality of smooth semi‐nonparametric models based on SNP densities is still lacking in general. However, empirical evidence from several studies on density estimation and survival analysis suggest that the use of standard asymptotic inference for MLE based on the final fit and ignoring the adaptive choice of the degrees of the SNP polynomials yield acceptable performance 9, 11. Accordingly, we base the calculation of standard errors and confidence intervals on the observed Fisher information matrix $I(\hat{\theta })$ with respect to $\hat{\theta }\equiv \hat{\theta }(K)$ with **K** being the SNP polynomial degrees of the final fit. Specifically, to obtain pointwise confidence for *C* *I* *F*_*j*_(*t*), we propose to first determine a Wald‐type confidence interval for the complementary log‐log (cloglog) transform of this quantity using the delta‐rule and then to transform this confidence interval back to the original scale. Operating on the cloglog‐scale is expected to improve the validity of the normal approximation and avoids having out of range confidence intervals. Of note, we also considered the usage of sandwich type robust variance estimator (e.g., equations (4.5.2) and (4.5.4) in 17) for the aforementioned calculations, but they did not lead to improved coverage in our simulation studies.

## 5 Between‐group comparisons of cumulative incidence functions

Doehler and Davidian suggested methods for comparing survival functions between two independent groups based on SNP estimates 10. A similar approach is possible for between‐group comparisons of CIFs. Without loss of generality, assume that the CIF corresponding to the first event type is of interest. The null hypothesis and alternative hypothesis are

$$\begin{array}{ccc} H_{0} & :CIF_{1}^{1}(\cdot )=CIF_{1}^{2}(\cdot ) & \\ H_{A} & :\left(CIF_{1}^{1}(\cdot )⩾CIF_{1}^{2}(\cdot )\;\;\text{or}\;\;CIF_{1}^{1}(\cdot )⩽CIF_{1}^{2}(\cdot )\right)\;\;\text{and}\;\;CIF_{1}^{1}(\cdot )\neq CIF_{1}^{2}(\cdot ) \end{array}$$

where superscripts denote the groups. A suitable test statistic is the integrated weighted difference (IWD):

(6) $$IWD=\int_{0}^{\tau }W(t)\left\{{\hat{CIF}}_{1}^{1}(t)-{\hat{CIF}}_{1}^{2}(t)\right\}dt$$

where *W*(·) is a positive weight function and *τ* is a suitably chosen time horizon. In principle, the weight function *W*(·) can be manipulated to make the test more sensitive to a specific alternative hypothesis, and for non‐parametric CIF estimates, *W*(·) also serves as a stabilization tool to down‐weight time points where the risk set is small and hence the test statistic unstable 18. Instability is less of an issue for parametric or SNP estimates. Thus, if we are not interested in any specific alternative hypothesis, we can simply set *W*(·)=1. Two possibilities for deriving the null distribution are available. First, one can estimate the variance of the test statistic based on *ad hoc* asymptotic inference and the delta‐rule and use this as the basis of a Wald‐type test. Alternatively, the null distribution and the resulting *p*‐value can be evaluated exactly without relying on a normal approximation by implementing a permutation test (or a Monte‐Carlo approximation to it). Details regarding the Wald‐type test as well as the permutation test are given in Section F of the Supporting Information.

## 6 Simulation study

**Simulation study for two event types (*J*=2) — Setting:** Several competing risks scenarios with right or interval censoring were investigated to compare the proposed SNP‐CIF estimation method to alternative parametric and nonparametric approaches in a series of simulations. All scenarios considered two competing events only, that is, *J*=2, and uncensored data were simulated either according to the mixture factorization (2) or a latent failure time model. For the scenarios based on the mixture factorization, we either chose a Weibull distribution for both conditional time to event distributions or a log‐normal distribution for the first event type and a SNP distribution with *K*=1 and a standard normal base density for the second event type, respectively. For simulation from a SNP distribution, we used rejection sampling as suggested in 19 and detailed in Appendix C of the Supporting Information. The corresponding marginal distribution of the first event type was chosen as 25% or 50%, respectively. The last scenario was based on two independent latent failure times *T*_1_ and *T*_2_ leading to $T=\min\left(T_{1},T_{2}\right)$ and $D=1I\left(T_{1}⩽T_{2}\right)+2I\left(T_{1}>T_{2}\right)$. We chose a mixture log‐normal distribution for *T*_1_ and a Weibull distribution for *T*_2_. Exact parametric choices for all scenarios are summarized in Table 1.

**Table 1:** Specification of the distribution of uncensored competing risks data for the scenarios investigated in the simulation study.

Mixture representation based scenario			
Scenario	*T*|*D*=1	*T*|*D*=2	*P*_1_ *%*
*2 Weibull*			
	$W\left(1^{-1},e^{-1}\right)$	$W\left(0.2^{-1},e^{-0.25}\right)$	25
*2 SNP stdnorm*			
	$LN\left(-0.1,1^{2}\right)$	$SN\left(-0.1,0.5,\frac{\pi }{9}\right)$	50
Latent failure times‐based scenario			
Scenario	*T*_1_	*T*_2_	*P*_1_ *%*
*Logmixturenorm + Weibull*			
	$0.3LN\left(-0.1,0.3^{2}\right)+0.7LN\left(0.7,0.1^{2}\right)$	$W\left(0.5^{-1},e^{1}\right)$	66

*Note*: *T*|*D*=*j* is the time to event distribution for event type *j* conditional on the occurrence of that event type. *P*_1_ is the marginal probability of event type 1. *T*_*j*_ is the independent latent failure time distribution associated with event type *j* used in the latent failure times‐based scenario. *W*(*σ* ^−1^,*e* ^*μ*^) refers to a Weibull distribution with shape *σ* ^−1^ and scale *e* ^*μ*^ and $LN\left(\mu ,\sigma^{2}\right)$ to a log‐normal distribution with mean *μ* and variance *σ* ^2^ for the log‐transformed data. *S* *N*(*μ*,*σ*,*ϕ*) is a random variable *T* satisfying logT=μ+σZ, where *Z* has a smooth semi‐nonparametric distribution with a standard normal base density and polynomial degree *K*=1 with a corresponding spherical coordinate *ϕ*.

For scenarios with independent right‐censoring, a censoring time *C* was simulated and the observed right‐censored data defined as $\left(T^{\prime }=\min\{T,C\},D^{\prime }=\Delta \times D\right)$, where Δ=1 if *T*⩽*C* and Δ=0 otherwise. The censoring distribution *C* was simulated as $C=\min\{t_{m},C_{E}\}$ where the maximum follow‐up duration *t*_*m*_ was chosen as the 50% or 90% quantile of the total survival time *T* and *C*_*E*_ was simulated according to an exponential distribution with rate *λ* chosen appropriately to ensure the desired overall right censoring probability of 65% or 35%, respectively. The parameters *t*_*m*_ and *λ* were determined via simulation using a large competing risks dataset of size *n*=10^6^ from the respective scenario. In the presentation of the simulation results, the two different right‐censoring schemes are denoted as ‘65% RC’ and ‘35% RC’, respectively.

For scenarios with independent interval censoring, we first determined the maximum follow‐up time *t*_*m*_ as the 50% or 90% quantile of the total survival time *T* as before. Interval censoring was then simulated as follows: If *t*_*m*_ corresponded to the 90% quantile (respectively median) of *T*, we split the interval [0,*t*_*m*_] into 10 (respectively 7) equally spaced sub‐intervals. For each observation, the corresponding observation process was defined as occurring at the sub‐interval cut‐points plus some observation‐specific normally distributed ‘noise’ with mean 0 and standard deviation equal to 1/5 of the sub‐interval length for time points other than 0 and *t*_*m*_. If a subject had an event before time *t*_*m*_, then their data was considered interval‐censored between the two adjacent time points of the observation process; otherwise, they were considered right‐censored at *t*_*m*_. In the presentation of the simulation results, the two different interval censoring schemes are denoted as ‘IC‐1’ (*t*_*m*_=90% quantile, 10 sub‐intervals) and ‘IC‐2’ (*t*_*m*_=50% quantile, 7 sub‐intervals), respectively.

Finally, the sample size was varied between 100 and 500 leading to 12 simulation settings with right censoring and 12 with interval censoring. Reported simulation results are based on 1000 simulated datasets per setting and maximum allowed polynomial degrees *K*_*max*_=3.

For the adaptive choice of the polynomial degrees and base density, the following information criteria were investigated: AIC, BIC*n*, BIC*d*, HQC*n*, and HQC*d*. As simulation results based on the sample size *n* and the number of events *d* were very similar, only results based on *n* are reported, and the best fits according to the different information criteria are denoted as SB‐AIC, SB‐BIC*n*, and SB‐HQC*n*. Competing methods included parametric and non‐parametric estimators. Parametric estimators were based on the mixture factorization assuming log‐normal or Weibull conditional time to event distributions that are available as a special case of our algorithm with polynomial degrees restricted to *K*=0. For right‐censored data, the standard nonparametric Aalen–Johansen estimator of the CIF was used and estimates for interval‐censored data were based on a nonparametric maximum likelihood estimator for bivariate distribution (*X*,*Y*) described in 20 and implemented in function computeMLE of the R package MLEcens 21. As the nonparametric maximum likelihood estimator can only assign mass to a finite number of disjoint intervals, we chose a unique representation by distributing the assigned mass uniformly across the respective intervals.

**Simulation study for two event types (*J*=2) — Results:** Detailed results for interval‐censored settings are provided here. Right‐censored settings are only summarized but detailed results are provided in Appendix D of the Supporting Information. Table 2 summarizes how frequently our method selected both the correct base density and the correct polynomial degrees of the corresponding conditional time to event distributions for mixture representation‐based scenarios. For the lower sample size of *n*=100, the average proportion of correctly chosen base densities and polynomial degrees was quite low, ranging from 32% for AIC to 58% for BIC*n*. For *n*=500, these proportions increased sharply and ranged from 60% for AIC to 91% for BIC*n* with an intermediate value of 80% for HQC*n*. Slightly higher proportions and a similar pattern were observed for right‐censored settings; for *n*=500 and HQC*n*, the proportion of correctly chosen models was 85% (Table 1 of Appendix D of the Supporting Information). The last two rows in Table 2 show the frequency with which fits to data from latent failure time models (which do not follow a SNP mixture model) used the maximum allowed polynomial degree of *K*=3. In general, AIC was more likely to exhaust the maximum complexity, although for right‐censored data this was not true for one right‐censored setting with *n*=500, which can be explained by the fact that AIC frequently chose a different base density compared with the other criteria (Table 1 of Appendix D of the Supporting Information).

**Table 2:** Frequency with which the proposed smooth semi‐nonparametric estimators based on AIC, BIC*n*, or HQC*n*, respectively, chose the correct base density and correct polynomial degrees for mixture representation based scenarios (first four rows).

		AIC		BIC*n*		HQC*n*	
Scenario	*n*	100	500	100	500	100	500
Frequency of correct base density and polynomial degrees							
*2 Weibull IC‐1*		343	650	649	977	488	888
*2 Weibull IC‐2*		162	496	447	769	286	657
*2 SNP stdnorm IC‐1*		471	657	677	970	597	875
*2 SNP stdnorm IC‐2*		305	585	528	915	436	787
Frequency that the selected model chose *K*_1_ = 3 or *K*_2_ = 3							
*Logmixturenorm + Weibull (LFT) IC‐1*		250	243	25	50	102	136
*Logmixturenorm + Weibull (LFT) IC‐2*		12	45	1	0	2	5

AIC, AkaikeŠ's information criterion; BIC*n*, Bayesian information criterion; HQC*n*, Hannan–Quinn's criterion; LFT, latent failure times.

For the other scenarios, the frequency with which the maximal allowed polynomial degree was chosen (i.e., *K*_1_=3 or *K*_2_=3) is reported (rows 5 and 6). All results are for scenarios with interval censoring.

All frequencies are based on 1000 simulated data sets per scenario.

IC‐1 refers to interval censoring with 10 sub‐intervals and right‐censoring at the 90% quantile.

IC‐2 refers to interval censoring with seven sub‐intervals and right‐censoring at the 50% quantile.

**Figure 1:**
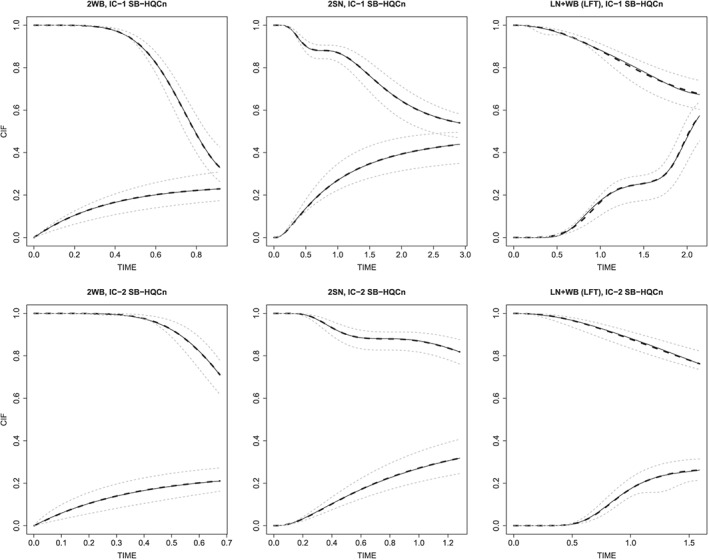
Simulation results for SB‐HQC*n* models for all scenarios with interval censoring and a sample size of *n*=500. *Note:* Bold lines show the true *C* *I* *F*_1_ for event type 1, and 1−*C* *I* *F*_2_ for event type 2 from time 0 to *t*_*m*_. Bold dashed lines show the corresponding point‐wise averaged fitted cumulative incidence functions across 1000 simulation runs. Light dashed lines show curves, resulting from the two simulation runs leading to the minimal and maximum average residual from the true curve based on 300 equally spaced time point from 0 to *t*_*m*_, that is, they display the worst observed under‐estimation and over‐estimation. From left to right are the scenarios: 2 weibull, 2 SNP stdnorm, logmixturenormal + Weibull (LFT). Top and bottom rows correspond to ‘IC‐1’ and ‘IC‐2’ scenarios, respectively.

The precision of CIF estimates was evaluated based on the integrated mean squared error (IMSE) from time 0 to *t*_*m*_, which was defined for event type *j* as

(7) $$IMSE_{j}=\frac{1}{N_{s}}\overset{N_{s}}{\underset{i=1}{\sum }}\left[\int_{0}^{t_{m}}{\left\{{\hat{CIF}}_{ji}(t)-CIF_{j}(t)\right\}}^{2}dt\right]$$

where *N*_*s*_ is the total number of simulated datasets, ${\hat{CIF}}_{ji}$ refers to the estimated CIF for event type *j* from the *i*th dataset of the considered simulation setting and *C* *I* *F*_*j*_ is the respective true CIF. Table 3 shows the relative IMSE of parametric and nonparametric methods compared with the SB‐HQC*n* model and absolute IMSE values for SNP‐based models for scenarios with interval censoring. If a parametric Weibull or log‐normal model was the true model for the respective CIF, SNP models based on HQC*n* were slightly outperformed by the true parametric model. If this was not the case, the SNP‐based model usually outperformed the parametric models, sometimes dramatically so. Moreover, our method consistently outperformed the non‐parametric estimator. Conclusions were similar for right‐censored data (Table 2 in Appendix D of the Supporting Information), although in this setting the non‐parametric estimator outperformed our estimator for one scenario. Regarding the investigated information criteria, BIC*n* often performed best, although for some complex right‐censored scenarios, AIC*n* performed considerably better. HQC*n*, the recommended information criterion in survival analysis 9, 10, provided intermediate performance and CIF estimates that resembled the truth quite closely (Figure 1). Furthermore, to see if the IMSE might be driven by single time regions, we also looked at the mean squared error at the specific time points *t*_*m*_ and *t*_*m*_/2. However, this leads to qualitatively identical conclusions to the results for the IMSE reported previously (data not shown).

**Table 3:** IMSE for different estimation methods for all scenarios with interval censoring.

	*n* = 100		*n* = 500	
Scenario	*C* *I* *F*_1_	*C* *I* *F*_2_	*C* *I* *F*_1_	*C* *I* *F*_2_
*2 Weibull IC‐1*				
LN/SB‐HQC*n*	**0.96** 0.008	**1.61** 0.049	**1.13** 0.015	**4.66** 0.178
WB/SB‐HQC*n*	**0.95** 0.006	**0.76** 0.016	**0.97** 0.005	**0.93** 0.013
NP/SB‐HQC*n*	**1.19** 0.010	**1.88** 0.045	**1.49** 0.021	**3.02** 0.089
SB‐AIC ×10^4^	8.71	3.72	1.53	0.62
SB‐BIC*n*×10^4^	8.33	3.16	1.47	0.56
SB‐HQC*n*×10^4^	8.54	3.43	1.51	0.58
*2 Weibull IC‐2*				
LN/SB‐HQC*n*	**0.95** 0.005	**1.02** 0.015	**0.98** 0.005	**1.19** 0.034
WB/SB‐HQC*n*	**0.96** 0.005	**0.90** 0.010	**0.98** 0.004	**0.81** 0.017
NP/SB‐HQC*n*	**1.22** 0.010	**1.78** 0.038	**1.45** 0.020	**3.36** 0.108
SB‐AIC ×10^4^	9.05	4.78	1.84	1.03
SB‐BIC*n*×10^4^	8.77	4.31	1.79	0.81
SB‐HQC*n*×10^4^	8.89	4.54	1.81	0.90
*2 SNP stdnorm IC‐1*				
LN/SB‐HQC*n*	**0.93** 0.013	**2.37** 0.065	**1.09** 0.019	**8.68** 0.325
WB/SB‐HQC*n*	**0.92** 0.013	**1.55** 0.031	**1.24** 0.027	**4.63** 0.156
NP/SB‐HQC*n*	**1.50** 0.027	**1.32** 0.018	**2.21** 0.056	**1.67** 0.032
SB‐AIC ×10^4^	17.61	11.18	3.12	2.17
SB‐BIC*n*×10^4^	16.73	11.57	2.98	2.10
SB‐HQC*n*×10^4^	17.19	11.31	3.04	2.13
*2 SNP stdnorm IC‐2*				
LN/SB‐HQC*n*	**0.92** 0.008	**1.25** 0.016	**0.99** 0.011	**2.24** 0.054
WB/SB‐HQC*n*	**0.97** 0.008	**1.23** 0.014	**1.19** 0.017	**2.53** 0.066
NP/SB‐HQC*n*	**1.30** 0.014	**1.22** 0.011	**1.58** 0.024	**1.43** 0.022
SB‐AIC ×10^4^	21.25	12.52	4.10	2.58
SB‐BIC*n*×10^4^	20.16	12.69	3.95	2.49
SB‐HQC*n*×10^4^	20.89	12.52	4.01	2.53
*Logmixturenorm + Weibull (LFT) IC‐1*				
LN/SB‐HQC*n*	**1.80** 0.043	**1.13** 0.025	**5.40** 0.166	**2.42** 0.060
WB/SB‐HQC*n*	**1.65** 0.041	**0.91** 0.011	**5.10** 0.148	**1.22** 0.013
NP/SB‐HQC*n*	**1.24** 0.013	**1.28** 0.017	**1.35** 0.016	**1.60** 0.036
SB‐AIC ×10^4^	17.06	14.88	3.62	2.75
SB‐BIC*n*×10^4^	16.86	14.09	3.77	2.69
SB‐HQC*n*×10^4^	16.94	14.46	3.64	2.67
*Logmixturenorm + Weibull (LFT) IC‐2*				
LN/SB‐*n*	**0.96** 0.004	**0.95** 0.011	**0.93** 0.005	**1.03** 0.011
WB/SB‐HQC*n*	**0.99** 0.005	**0.89** 0.010	**1.12** 0.009	**0.88** 0.014
NP/SB‐HQC*n*	**1.26** 0.011	**1.38** 0.019	**1.53** 0.022	**1.80** 0.039
SB‐AIC ×10^4^	20.23	15.67	3.74	2.77
SB‐BIC*n*×10^4^	16.86	14.09	3.77	2.69
SB‐HQC*n*×10^4^	16.94	14.46	3.64	2.67

*Note*:  LN/SB‐HQC*n*, WB/SB‐HQC*n*, and NP/SB‐HQC*n* are the ratios (in bold) of the integrated mean squared error (IMSE) of the parametric log‐normal, Weibull, and the nonparametric models, respectively, versus the SB‐HQC*n* model with corresponding bootstrap standard errors. For each scenario, the last three rows give IMSE values of smooth semi‐nonparametric‐based models for all information criteria. IC‐1 (IC‐2) refers to interval censoring with 10 (7) sub‐intervals and right‐censoring at the 90% (50%) quantile.

Table 4 and Table 2 in Appendix D of the Supporting Information give point‐wise Monte Carlo coverage probabilities of nominal Wald‐type 95% confidence interval for all CIF estimators at two selected time points, 0.5*t*_*m*_ and *t*_*m*_, for interval‐censored and right‐censored scenarios, respectively. All confidence intervals were based on the complementary log‐log transformation, and for SNP‐based estimates, they used *ad hoc* asymptotic inference as described in Section 4.3. Our model based on HQC*n* showed some undercoverage, but observed coverage was larger than 90% for 45/48 reported values for interval‐censored settings and 43/48 reported values for right‐censored settings with the lowest observed coverage being 82%. Models based on BIC*n* and AIC*n* showed comparable although overall slightly worse coverage.

**Table 4:** Observed coverage probabilities of nominal 95% CI for the cumulative incidence functions at times 0.5*t*_*m*_ and *t*_*m*_ for interval‐censored scenarios.

	*n* = 100				*n* = 500			
Time point	0.5*t*_*m*_		*t*_*m*_		0.5*t*_*m*_		*t*_*m*_	
Scenario	*C* *I* *F*_1_	*C* *I* *F*_2_	*C* *I* *F*_1_	*C* *I* *F*_2_	*C* *I* *F*_1_	*C* *I* *F*_2_	*C* *I* *F*_1_	*C* *I* *F*_2_
*2 Weibull IC‐1*								
LN	94.1	63.1	95.2	81.6	92.3	8.5	95.0	39.0
WB	94.6	95.9	94.5	94.3	95.5	96.1	94.7	94.6
SB‐AIC	94.6	82.6	93.6	92.4	94.8	90.8	95.1	93.4
SB‐BIC*n*	94.0	86.3	94.2	92.9	95.5	95.1	94.7	94.5
SB‐HQC*n*	94.4	83.9	93.8	93.1	95.2	93.2	94.8	94.2
*2 Weibull IC‐2*								
LN	95.3	95.5	95.8	94.9	94.6	79.9	95.2	94.1
WB	95.2	95.6	95.7	94.4	94.4	95.7	95.5	94.7
SB‐AIC	94.3	89.4	95.5	93.6	94.4	86.7	95.6	94.7
SB‐BIC*n*	94.5	94.5	95.8	94.2	94.6	90.8	95.5	94.5
SB‐HQC*n*	94.3	93.2	95.7	94.2	94.8	89.5	95.5	94.7
*2 SNP stdnorm IC‐1*								
LN	94.5	91.3	93.9	64.4	95.1	69.5	94.3	5.4
WB	95.0	85.2	94.0	80.5	92.3	37.0	95.1	42.0
SB‐AIC	94.0	96.1	93.0	94.3	95.3	94.0	95.3	95.8
SB‐BIC*n*	94.5	94.9	93.6	93.5	95.8	94.0	95.7	95.6
SB‐HQC*n*	94.1	95.8	93.4	94.1	95.7	93.9	95.6	95.6
*2 SNP stdnorm IC‐2*								
LN	94.9	94.7	94.3	95.6	92.3	82.1	94.3	94.1
WB	94.4	91.3	93.9	95.2	86.9	48.6	94.7	93.8
SB‐AIC	93.7	94.5	94.5	95.2	92.5	95.0	95.8	94.7
SB‐BIC*n*	94.0	94.4	94.2	95.4	93.3	94.9	95.5	94.5
SB‐HQC*n*	93.5	94.4	94.4	95.5	92.6	94.9	95.5	94.5
*Logmixturenorm + Weibull (LFT) IC‐1*								
LN	89.5	85.6	33.3	96.5	44.5	72.9	0.1	94.2
WB	84.4	93.6	56.0	95.6	22.6	93.1	3.8	91.9
SB‐AIC	92.4	94.1	94.8	94.5	86.8	93.9	94.2	93.7
SB‐BICn	92.3	93.5	94.1	95.0	83.0	94.4	92.9	93.1
SB‐HQCn	92.1	93.7	94.3	94.3	86.4	94.5	93.4	93.3
*Logmixturenorm + Weibull (LFT) IC‐2*								
LN	94.7	93.7	95.4	95.4	96.6	91.6	95.6	95.7
WB	96.9	96.6	95.4	94.5	93.0	95.9	95.6	94.8
SB‐AIC	93.3	94.3	95.3	94.1	93.0	93.7	95.6	95.0
SB‐BIC*n*	94.2	94.5	95.3	94.5	95.1	92.5	95.6	95.2
SB‐HQC*n*	93.7	94.4	95.3	94.2	94.2	92.8	95.6	95.1

*Note*:  LN and WB refer to parametric log‐normal and Weibull models. SB‐AIC, SB‐BIC*n*, and SB‐HQC*n* refer to smooth semi‐nonparametric‐based models using different information criteria. Estimated Monte Carlo standard error of observed coverage ≈0.69*%*. IC‐1 refers to interval censoring with 10 sub‐intervals and right‐censoring at the 90% quantile. IC‐2 refers to interval censoring with seven sub‐intervals and right‐censoring at the 50% quantile.

Parametric models usually performed well if the parametric model reflected the truth but demonstrated dramatic undercoverage in some other settings. Coverage for the non‐parametric estimator in the presence of interval censoring was not evaluated as confidence intervals are not provided by the used software and the estimator has non‐standard asymptotic properties 21. For right‐censored settings, the nonparametric estimator performed well with observed coverage of at least 93.5*%* across all scenarios except for two very low observed values for the Weibull mixture scenario with *n*=100. The latter is potentially due to the very low observed frequency of events of type 2 before time 0.5*t*_*m*_ in these settings.

Bootstrap‐based confidence interval for SNP‐estimators might lead to improved coverage as demonstrated in the survival setting in 10, but we did not explore this in our simulations as the estimation algorithm is computer intensive. Specifically in our simulation, median (mean) computing times for CIF estimation based on HQC*n* with a sample size of *n*=500 were 91 (88) and 127 (212) seconds across the six interval‐censored and right‐censored settings, respectively (on a Windows PC with an Intel Core i7‐3770 CPU and 10GB Ram).

**Additional simulation study for three event types (*J*=3):** An additional simulation study was conducted for a scenario with three competing risks based on SNP distributions having standard normal base densities and polynomial degrees of 0, 2, and 3, respectively. The simulation scenario and results are detailed in Appendix E of the Supporting Information. A lower frequency of choosing the correct base densities and polynomial degrees (reported in Table 5 of the Supporting Information) than for the simpler scenarios with *J*=2 and true *K*_*max*_=1 reported in Table 2 was noted. This is not unexpected and a similar effect could already be seen when contrasting results from the competing risks settings with *J*=2, shown in Table 2, against results from the less complex survival settings investigated by Doehler and Davidian 10. However, performance in terms of the integrated mean‐squared error (Table 6 of the Supporting Information) remained superior to the parametric and non‐parametric alternatives and true coverage of nominal 95% confidence intervals (Table 7 of the Supporting Information) was also generally acceptable acknowledging some mild undercoverage.

**Figure 2:**
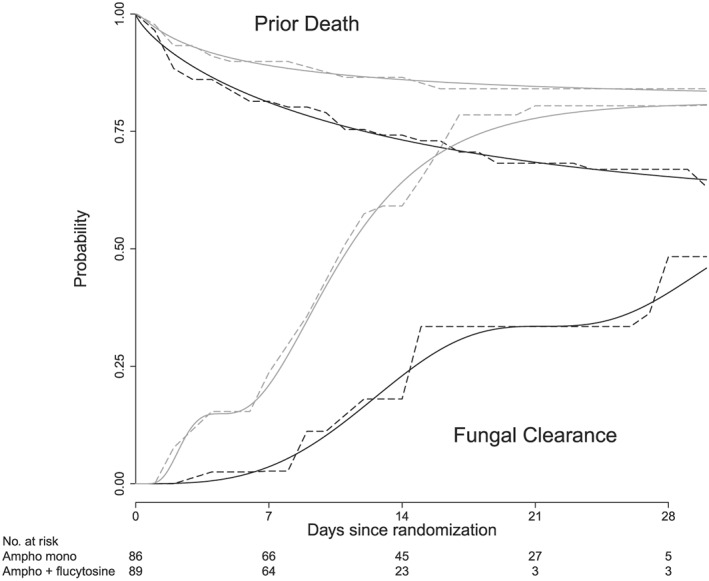
Estimated cumulative incidence function for the time to fungal clearance (increasing lines) and one minus cumulative incidence function for prior death (decreasing lines) by treatment arm for the smooth semi‐nonparametric‐based estimator using HQC*n* and the nonparametric estimator. Black lines correspond to amphotericin B monotherapy, gray lines to amphotericin B plus flucytosine. Solid lines refer to smooth semi‐nonparametric estimates, dashed lines to nonparametric estimates. As the nonparametric maximum likelihood estimator for interval‐censored outcomes can only assign mass to a finite number of disjoint intervals, we chose a unique representation by distributing the assigned mass uniformly across the respective intervals.

**Type I error for the between‐group comparisons of cumulative incidence functions:** We used our main simulation study (with *J*=2) to investigate whether the asymptotic IWD‐based test proposed in Section 5 protects the type I error at the nominal 5*%* significance level. Specifically, we used the 1000 simulation runs from each scenario to form 500 pairs of two‐group comparisons under the null hypothesis that both groups are equal. According to normal plots *(provided in Figure 3 of Section G of the Supporting Information*), the resulting 500 IWD statistics using the simple weight function *W*(·)=1 had an approximate normal distribution for all scenarios and both event types. However, estimates of its standard error calculated based on *ad hoc* asymptotic inference and the delta‐rule tended to be too large. The consequence of this was that the simulated type‐I errors were conservative, that is, below 5*%* for all scenarios and event types, but they were generally too small with 8/20(40*%*) and 8/20(40*%*) of values <1*%* for simulations with *n*=100 and *n*=500 per group, respectively. *Quantile plots of the p‐values against the target uniform distribution are provided in Figure 2 of Section G of the Supporting Information*. Based on these results, we suggest using the proposed (exact) permutation test instead acknowledging the higher computational burden.

## 7 Analysis of a trial in cryptococcal meningitis

We used data from a randomized controlled clinical trial comparing antifungal therapies in HIV‐infected patients with cryptococcal meningitis 22, and for simplicity, we restricted attention to two of the three randomized interventions: Amphotericin B monotherapy (a frequent treatment in Asia) and combination therapy with amphotericin B and flucytosine (an expensive treatment recommended by treatment guidelines). The competing risks endpoint of interest here was the time from randomization to clearance of quantitative fungal counts in cerebrospinal fluid (beneficial event of interest) or death without prior fungal clearance (harmful competing event) during a follow‐up period of 30 days. Quantitative fungal counts were only measured weekly, and hence, the time of fungal clearance was only known to have occurred between the last positive and the first zero measurement, that is, it is interval‐censored. The date of death was known exactly, and the time to death was treated as interval‐censored during the corresponding 24‐h interval. Patients without an event were right‐censored at the time of their last positive fungal count measurement. Of note, because of interval‐censoring, the data could in principle have missed unobserved fungal clearances for patients who died without observed fungal clearance. However, based on the available quantitative fungal count measurements for these patients, this seemed highly unlikely for almost all cases. The dataset contained 175 patients: 103 reached fungal clearance, 43 died without prior clearance, and 29 were right‐censored.

Cumulative incidence function estimates are displayed in Figure 2 and show good agreement between the SNP‐based estimator using HQC*n* and the nonparametric estimator. The figure suggest both a faster time to clearance and a lower risk of prior death for the combination therapy. For a formal comparison, we calculated *p*‐values for the IWD‐based test statistic introduced in Section 5 using a trivial weight function and *ad hoc* asymptotic inference or, alternatively, a permutation test (approximated using 1000 Monte Carlo samples). *P*‐values were *p*<0.001 (asymptotic and permutation test) for the comparison of CIFs for fungal clearance and *p*=0.020 (asymptotic) or *p*=0.026 (permutation test) for prior death, respectively.

## 8 Discussion

In this paper, we have developed a new SNP CIF estimation technique for competing risks data. The underlying statistical model is specified via a mixture factorization of the joint distribution of the event type and time. The time to event distributions conditional on the event type are modeled using SNP densities. One key strength of the approach is that it can handle arbitrary censoring and truncation while producing smooth estimates with only mild parametric assumptions. We further suggested a stepwise forward procedure for model estimation. This procedure returns parametric mixture log‐normal or Weibull models as starting points, and gradually increases model complexity until the optimal model based on some information criterion is found.

In an extensive simulation study, we demonstrated that the proposed approach provides smooth estimates of the CIF over the observed follow‐up period that are frequently more accurate than parametric and nonparametric alternatives even under heavy censoring. The simulations also support the use of ‘ad hoc’ asymptotic inference to derive confidence intervals although some undercoverage was observed. This is in accordance with other statistical areas such as density estimation 11, time‐series analysis 19, random effects modeling 23, and survival analysis 9, 10 where the SNP approach has been successfully applied. Of note, the CIF is identifiable over the observed follow‐up period, but the marginal event probabilities, *P*(*D*=*j*), are not identifiable in a non‐parametric sense. However, the proposed model is weakly identifiable for each SNP polynomial degree because of the additional parametric assumptions. As shown in our simulations, this weak identifiability does not appear to corrupt estimation of the CIF over the observed time range. In principle, our model also allows for the extrapolation of the CIF beyond the observed follow‐up period and the estimation of marginal event probabilities. However, we caution against this usage of our method except if there is minimal censoring and these probabilities are truly identifiable from the data. Not unexpectedly, the simulations also revealed that it is more difficult to identify the true data‐generating SNP models when the number of competing risks increased from two to three. However, in the setting of three competing risks, our proposed method remained competitive compared with parametric and non‐parametric alternatives in terms of the accuracy of the estimated CIFs.

An extension of the proposed model to the regression setting is straightforward by including covariates into the accelerated failure time sub‐model (4) and the multinomial logistic sub‐model (3). This leads to a flexible regression model for competing risks data that can handle arbitrary censoring and truncation, which has been investigated in detail in the PhD thesis of the first author 24. Of note, in common with other proposed competing risks models based on the mixture factorization 12, 14, the model has interpretational and identifiability issues, especially if there is insufficient follow‐up relative to the timing of the events.

Despite the strong performance of models based on SNP densities in simulation studies across statistical areas, a formal theory that would allow to derive asymptotically valid confidence intervals and hypothesis tests is still largely lacking. This provides a potential area for future research that could benefit from recent advances in asymptotic theories for the broader class of sieve MLE estimators 25, 26. Moreover, while the proposed algorithm worked well, it is computationally intensive. The development of faster and more robust algorithms would allow for the exploration of bootstrap methods to improve the coverage of confidence intervals and of models with larger polynomial degrees *K*, which could further improve estimation if the true CIFs are very irregular. Finally, this paper focused on the CIF as the target of inference. However, competing risks models based on modeling the cause‐specific hazards are also popular 27. One approach to applying SNP densities to cause‐specific hazards models would be to extend the SNP Cox proportional hazards model developed by 9.

## 9 Software and datasets

An implementation of the proposed estimating algorithm by the first author in the statistical software R 16 has been uploaded to GitHub: https://github.com/nguyenducanhvn101087/R_SNP_Competing_Risks We thank our clinical colleagues 22 for allowing us to make the cryptococcal meningitis dataset discussed in Section 7 freely available. This dataset and corresponding analysis code has also been uploaded to the same GitHub location.

## Supporting information



Supplementary material for Smooth Semi‐nonparametric (SNP) Estimation of the Cumulative Incidence Function
