# Supplementary material for: Smooth semi‐nonparametric (SNP) estimation of the cumulative incidence function
Source: Stat Med. 2017 May 23;36(18):2921–34. doi: 10.1002/sim.7331 (PMC5518232; doi:10.1002/sim.7331)
Supplement: Supplementary file 1 — Supplementary material for Smooth Semi‐nonparametric (SNP) Estimation of the Cumulative Incidence Function [file SIM-36-2921-s001.zip › snp_cif_est_webappend_sm_Final_Rev_DA.pdf]

Supplementary Material

Statistics in Medicine

(www.interscience.wiley.com)

Supplementary material for Smooth Semi-nonparametric (SNP) Estimation of the Cumulative Incidence Function

Anh Nguyen Duc[a]*and Marcel Wolbers[a,b]

A. Illustration of the Stepwise Forward Algorithm

A schematic of the stepwise forward algorithm introduced in Section 4.2 of the main paper for 2 competing risks ($J = 2$); $K_{\max} = 2$ and AIC as the information criterion is given in Figure 1. In this example, the final model would choose $K_1 = 0$ and $K_2 = 2$. The final forward step from $(K_1 = 1,\ K_2 = 2)$ to $(K_1 = 2,\ K_2 = 2)$ is not allowed as AIC at $(K_1 = 1,\ K_2 = 2)$ is already worse than AIC at the previous step

Figure 1. Adaptive greedy stepwise forward selection of polynomial degrees K_1 and K_2 using AIC

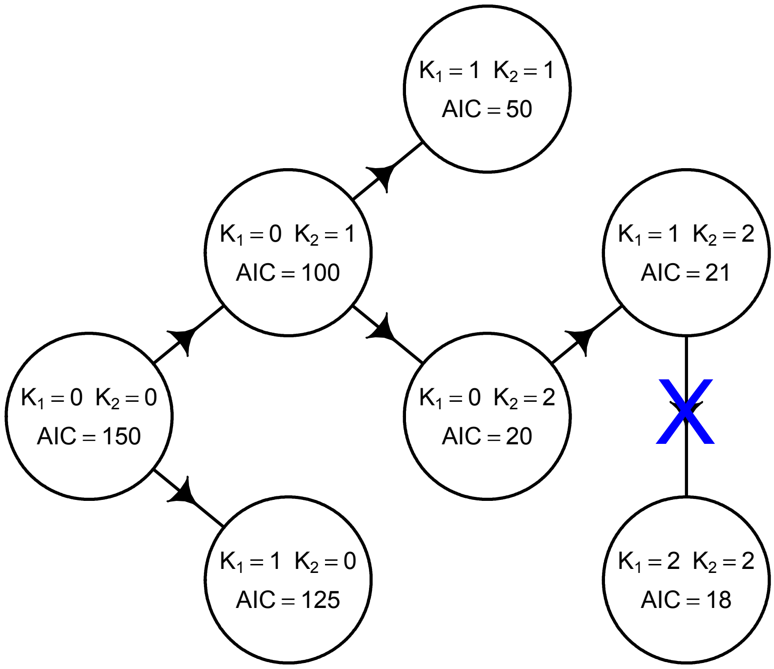

[a] *Oxford University Clinical Research Unit, Wellcome Trust Major Overseas Programme, Ho Chi Minh City, Viet Nam.*
[b] *Centre for Tropical Medicine, Nuffield Department of Medicine, University of Oxford, Oxford, UK.*
* *Correspondence to: Marcel Wolbers. E-mail: nguyenducanhvn101087@gmail.com*

B. Determination of Initial Values for the Stepwise Forward Algorithm

This section describes in detail how initial values for the parameters are set at each step of the stepwise forward algorithm described in Section 4.2 of the main paper.

B.1. Starting values for the parametric mixture factorization model (step 0)

In the parametric setting the conditional time-to-event distribution for each event type is specified as $\log(T \mid D = j) = \mu_j + \sigma_j Z$ leading to a log-normal model for $T \mid D = j$ if Z has a standard normal base density and to a Weibull model if $\exp(Z)$ has a standard exponential distribution. Hence, initial values for μ_j and σ_j can be obtained with standard software for fitting parametric accelerated failure time models (AFT). Specifically, we used the R function `survreg` from package `survival` [1] which supports both log-normal and Weibull models.

All observations with an observed event of type j were included and observations with other observed event types were excluded from the respective AFT. Right-censored observations (for which the event type is unknown) were included but downweighted. Specifically, a right-censored observation at time t received a weight corresponding to a crude estimate of the probability of ultimately experiencing event type j given by $P(D = j \mid T > t)$. Note that $P(D = j|T > t) = \dfrac{P(D = j, T > t)}{\sum_{k=1}^{J} P(D = k, T > t)}$ where $P(D = j, T > t) = P(D = j) - P(D = j, T \leq t)$ was approximated as follows: $P(D = j, T \leq t)$ was estimated based on the Nelson-Aalen estimator of the CIF (as implemented in the R function `cuminc` from package `cmprsk` [2]) and a crude estimate of the marginal event probabilities $P(D = j)$ was obtained by normalizing the values of the CIF estimates of all event types at the last observed time point such that their normalized sum was 1. For interval-censored observations, the event type is known, and they were included as interval-censored observations with weight 1 in the AFT. However, for simplicity, we used the mid-point of the interval as the exact event time for the sake of computing the weights in this case.

As suggested by [3] for survival analysis, in addition to using the starting values $(\mu_j, \sigma_j)_{j=1,\ldots,J}$ from the AFT models, we also used $(\mu_j \pm \sigma_j/2, \sigma_j)_{j=1,\ldots,J}$ as additional sets of starting values.

To get initial values for $\gamma_j, j = 1, \ldots, J-1$, we optimized the full log-likelihood with respect to these parameters while fixing $(\mu_j, \sigma_j)_{j=1,\ldots,J}$ at each set of starting values mentioned above. This sub-optimization itself requires initial values for $\gamma_j, j = 1, ..., J-1$, which were derived from the crude estimates of $P(D = j)$ described above exploiting that $\gamma_j = \log\left\{\frac{P(D=j)}{P(D=J)}\right\}$.

For the sake of determining starting values, left-truncation is currently ignored. In principle, it would be possible to extend the heuristic techniques outlined above to this setting but current R implementations for parametric AFT and nonparametric CIF estimation do not support left-truncation which would complicate implementation.

B.2. Starting values for the intermediate step (step 1)

At an intermediate step which increases $(K_1, \ldots, K_j, \ldots, K_J)$ to $(K_1, \ldots, K_j + 1, \ldots, K_J)$, initial values for γ_j and all parameters related to conditional survival distributions for event types other than j were set to the corresponding MLE values from the previous step.

To get starting values for survival parameters related to event type j, a grid of starting values was chosen for the spherical coordinates ϕ_{K_j+1} characterizing the polynomial coefficients as suggested by [3]. Specifically, we used the grid $\{-1.5, -1.3, ..., 1.3, 1.5\}$ for $K_j + 1 = 1$ and $\{-1.5, -0.5, 0.5, 1.5\}^{K_j+1}$ for $K_j + 1 > 1$ in the simulation study but the implementation allows to also specify alternative grids. A relatively large number of starting values is chosen because the log-likelihood is expected to be multi-modal with respect to the spherical coordinates.

For each initial value of ϕ_{K_j+1}, μ_j and σ_j were updated to correspond to the new SNP distribution such that the first two moments of $\log\{T \mid D = j\}$ remained unchanged compared to the fit from the previous step. Of note, these moments

are given by:

$$\begin{aligned} \mathrm{E}\left(\log T \mid D=j\right) &= \mu_j + \sigma_j \mathrm{E}\left(Z_j\right) \\ \mathrm{Var}\left(\log T \mid D=j\right) &= \sigma_j^2 \mathrm{Var}\left(Z_j\right) \end{aligned} \tag{1}$$

C. Simulation of Random Variables from a SNP Distribution

This section gives a univariate interpretation of Section 3 in [4] which discussed how to simulate a random variable having density $h(z) = \left(\sum_{i=0}^{K} a_i z^i\right)^2 \psi(z)$ based on the rejection method. The rejection method requires an upper envelope $b(z)$ with $h(z) \leq b(z)$. To define this envelope, first expand the polynomial $\left(\sum_{i=0}^{K} a_i z^i\right)^2$ to an explicit polynomial of degree $2K$ of the form $\sum_{i=0}^{2K} d_i z^i$. The envelope is then defined as $b(z) = \sum_{i=0}^{2K} |d_i| \, |z|^i \, \psi(z)$. Depending on the chosen base density, the process proceeds as follows.

C.1. Simulation for the standard normal base density

The envelope function is $b(z) = \left(\sum_{i=0}^{2K} |d_i| \, |z|^i\right) \varphi(z) = \frac{1}{\sqrt{2\pi}} \sum_{i=0}^{2K} |d_i| \, \frac{\Gamma\left(\frac{i+1}{2}\right)}{2^{\frac{1-i}{2}}} \chi(i+1, |z|)$. This is a weighted sum of chi-densities $\chi(i+1, |z|) = \frac{2^{\frac{1-i}{2}}}{\Gamma\left(\frac{i+1}{2}\right)} |z|^i \, e^{-z^2/2}$ with $i+1$ degrees of freedom. The chi-density describes the distribution of the square root of a random variable with a chi-squared distribution. The weights are then $u_i = \frac{1}{\sqrt{2\pi}} |d_i| \, \frac{\Gamma\left(\frac{i+1}{2}\right)}{2^{\frac{1-i}{2}}}, \ i = 0, \ldots, 2K$. Next we get normalized weights as $w_i = \frac{u_i}{\sum_{j=0}^{2K} u_j}$. Then the simulation proceeds as follows:

- Step 1: Use the multinomial distribution with cell probabilities w_i $(i = 1, \ldots, 2K+1)$ to randomly select a number in $\{1, ..., 2K+1\}$ as the degrees of freedom.
- Step 2: Simulate a chi-distributed number by taking the square-root of the corresponding chi-square distributed number having the above degree of freedom. Call this number z.
- Step 3: Randomly assign a sign to z with the probability of a negative or positive sign, respectively, set to 0.5. Denote the new number by v.
- Step 4: Generate u from the uniform distribution on $[0, 1]$. If $u > h(v)/b(v)$ return to step 1. Otherwise, accept v.

C.2. Simulation for the exponential base density

The envelope function is $b(z) = \left(\sum_{i=0}^{2K} |d_i| \, z^i\right) \exp(-x) = \sum_{i=0}^{2K} |d_i| \, \Gamma(i+1) G(1, i+1, z)$. This is a weighted sum of gamma densities G whose shape and scale parameters are 1 and $i+1$, respectively. The weights are then $u_i = |d_i| \, \Gamma(i+1), \ i = 0, \ldots, 2K$. Next we get the normalized weights as $w_i = \frac{u_i}{\sum_{j=0}^{2K} u_j}$. Then the simulation proceeds as for the normal base density, except that in step 2 a Gamma-distributed random variable is generated, step 3 is omitted, and $v = u$ is used in step 4.

D. Simulation Results for CIF Estimation with Only Right Censoring

Table 1. Frequency with which the proposed SNP estimators based on AIC, BICn or HQCn, respectively, chose the correct base density and correct polynomial degrees for mixture representation based scenarios (first 4 rows). For the other scenarios, the frequency with which the maximal allowed polynomial degree was chosen (i.e. $K_1 = 3$ or $K_2 = 3$) is reported (rows 5 and 6). All results are for scenarios with only right censoring.

		AIC		BICn		HQCn	
Scenario	n	100	500	100	500	100	500
Frequency of correct base density and polynomial degrees							
2 Weibull 35% RC		508	677	730	983	636	888
2 Weibull 65% RC		291	607	464	854	369	786
2 SNP stdnorm 35% RC		513	631	758	946	656	859
2 SNP stdnorm 65% RC		373	702	571	934	507	859
Frequency that the selected model chose $K_1 = 3$ or $K_2 = 3$							
Logmixturenorm + weibull (LFT) 35% RC		300	234*	76	411*	177	389*
Logmixturenorm + weibull (LFT) 65% RC		28	148	0	1	3	36

All frequencies are based on 1000 simulated data sets per scenario.
* Number of SB models based on AIC, BICn and HQCn with standard normal base densities: 100, 460 and 460, respectively.

Table 2. Integrated mean squared error (IMSE) for different estimation methods for all scenarios with only right censoring.

	$n = 100$		$n = 500$	
Scenario	CIF_1	CIF_2	CIF_1	CIF_2
2 Weibull 35% RC				
LN/SB-HQCn	**0.97** 0.009	**1.76** 0.057	**1.21** 0.017	**5.79** 0.229
WB/SB-HQCn	**0.97** 0.006	**0.84** 0.014	**1.00** 0.003	**0.95** 0.009
NP/SB-HQCn	**1.09** 0.008	**1.25** 0.021	**1.13** 0.008	**1.51** 0.033
SB-AIC $\times 10^4$	7.95	3.36	1.69	0.56
SB-BICn $\times 10^4$	7.76	3.17	1.65	0.50
SB-HQCn $\times 10^4$	7.84	3.26	1.66	0.52
2 Weibull 65% RC				
LN/SB-HQCn	**0.93** 0.007	**1.07** 0.013	**1.00** 0.010	**1.54** 0.037
WB/SB-HQCn	**0.99** 0.005	**0.92** 0.009	**1.01** 0.004	**0.95** 0.011
NP/SB-HQCn	**1.09** 0.007	**1.14** 0.011	**1.10** 0.007	**1.23** 0.017
SB-AIC $\times 10^4$	8.70	4.15	1.69	0.78
SB-BICn $\times 10^4$	8.52	4.01	1.69	0.75
SB-HQCn $\times 10^4$	8.60	4.08	1.69	0.76
2 SNP stdnorm 35% RC				
LN/SB-HQCn	**0.95** 0.014	**2.53** 0.082	**1.12** 0.019	**8.62** 0.363
WB/SB-HQCn	**0.96** 0.017	**1.54** 0.037	**1.34** 0.038	**4.41** 0.169
NP/SB-HQCn	**1.17** 0.016	**1.08** 0.013	**1.25** 0.021	**1.12** 0.014
SB-AIC $\times 10^4$	17.12	10.86	3.04	2.19
SB-BICn $\times 10^4$	16.67	10.56	2.95	2.19
SB-HQCn $\times 10^4$	16.94	10.66	2.99	2.18
2 SNP stdnorm 65% RC				
LN/SB-HQCn	**0.93** 0.008	**1.24** 0.015	**1.04** 0.010	**2.31** 0.055
WB/SB-HQCn	**0.99** 0.008	**1.22** 0.014	**1.26** 0.018	**2.63** 0.070
NP/SB-HQCn	**1.10** 0.009	**1.05** 0.005	**1.13** 0.010	**1.08** 0.007
SB-AIC $\times 10^4$	20.72	13.85	4.10	2.64
SB-BICn $\times 10^4$	19.95	13.89	4.03	2.63
SB-HQCn $\times 10^4$	20.31	13.87	4.06	2.63
Logmixturenorm + weibull (LFT) 35% RC				
LN/SB-HQCn	**1.41** 0.030	**1.08** 0.022	**4.19** 0.131	**1.64** 0.038
WB/SB-HQCn	**1.30** 0.027	**0.88** 0.011	**4.04** 0.125	**0.79** 0.016
NP/SB-HQCn	**0.83** 0.014	**1.04** 0.015	**0.78** 0.017	**0.73** 0.021
SB-AIC $\times 10^4$	21.69	14.76	4.02	2.89
SB-BICn $\times 10^4$	23.35	14.21	6.92	4.10
SB-HQCn $\times 10^4$	22.21	14.46	4.69	3.95
Logmixturenorm + weibull (LFT) 65% RC				
LN/SB-HQCn	**0.97** 0.004	**0.95** 0.011	**0.97** 0.003	**1.06** 0.012
WB/SB-HQCn	**1.01** 0.006	**0.89** 0.011	**1.16** 0.011	**0.87** 0.015
NP/SB-HQCn	**1.06** 0.005	**1.16** 0.013	**1.06** 0.005	**1.12** 0.016
SB-AIC $\times 10^4$	18.43	14.13	3.65	2.94
SB-BICn $\times 10^4$	23.35	14.21	6.92	4.10
SB-HQCn $\times 10^4$	22.21	14.46	4.69	3.95

Note: LN/SB-HQCn, WB/SB-HQCn and NP/SB-HQCn are the ratios (in bold) of the IMSE of the parametric log-normal, Weibull and the nonparametric models, respectively, versus the SB-HQCn model with corresponding bootstrap standard errors. For each scenario, the last 3 rows give IMSE values of SNP-based models for all information criteria.

Table 3. Observed coverage probabilities of nominal 95% CI for the CIFs at times $0.5t_m$ and t_m for right censored scenarios.

	$n = 100$				$n = 500$			
Time point	$0.5t_m$		t_m		$0.5t_m$		t_m	
Scenario	CIF_1	CIF_2	CIF_1	CIF_2	CIF_1	CIF_2	CIF_1	CIF_2
2 Weibull 35% RC								
LN	95.3	62.9	95.5	80.9	92.9	12.0	90.4	33.9
WB	94.8	93.4	96.2	94.9	93.9	95.5	93.8	95.2
NP	94.3	55.7	96.1	95.5	93.5	96.4	93.3	95.2
SB-AIC	94.2	85.1	96.3	92.1	93.7	92.1	93.8	94.4
SB-BICn	94.2	87.1	96.3	92.6	94.1	94.9	93.8	95.1
SB-HQCn	94.0	86.3	96.3	92.4	94.1	93.3	93.7	94.8
2 Weibull 65% RC								
LN	95.6	92.9	96.6	95.0	94.5	75.0	93.5	93.6
WB	94.8	95.3	96.0	94.9	93.5	95.9	94.7	95.3
NP	94.9	56.4	95.9	94.4	94.3	95.6	94.9	95.2
SB-AIC	94.2	92.5	96.3	94.4	94.1	90.4	95.0	95.5
SB-BICn	95.2	93.9	96.2	94.6	94.0	91.8	94.9	95.4
SB-HQCn	94.5	92.8	96.3	94.6	93.9	91.4	94.9	95.5
2 SNP stdnorm 35% RC								
LN	93.8	92.7	94.4	61.3	93.3	69.6	94.0	6.3
WB	93.9	85.9	93.9	82.8	87.2	27.2	94.2	48.4
NP	95.1	96.1	94.4	95.3	95.5	94.1	94.9	95.0
SB-AIC	92.5	95.1	94.5	94.1	94.9	94.4	94.4	93.9
SB-BICn	92.8	94.6	94.6	94.0	95.1	94.1	94.4	93.3
SB-HQCn	92.6	94.9	94.4	94.3	95.1	94.4	94.3	93.6
2 SNP stdnorm 65% RC								
LN	95.5	92.0	94.8	95.4	90.1	82.7	92.6	96.0
WB	93.7	88.5	94.7	95.0	85.1	41.9	92.8	93.7
NP	96.1	94.9	94.9	95.4	94.3	93.5	93.9	95.6
SB-AIC	93.7	94.7	95.3	95.1	93.4	94.0	93.5	95.6
SB-BICn	94.6	93.6	95.1	95.5	93.6	93.9	93.6	95.6
SB-HQCn	94.4	94.2	94.9	95.3	93.6	93.9	93.6	95.7
Logmixturenorm + weibull (LFT) 35% RC								
LN	87.1	85.7	34.3	97.0	42.1	61.9	0.2	94.5
WB	81.5	94.0	56.2	95.1	22.6	94.1	6.0	91.5
NP	95.7	96.1	94.3	95.6	93.9	95.6	93.8	94.5
SB-AIC	91.0	92.1	84.5	94.6	88.3	93.8	90.5	93.2
SB-BICn	88.2	92.0	79.8	94.8	76.3	86.2	70.2	92.6
SB-HQCn	90.2	92.2	82.0	94.8	86.6	87.2	83.1	93.0
Logmixturenorm + weibull (LFT) 65% RC								
LN	95.1	93.6	95.6	97.0	94.8	86.4	95.3	94.7
WB	97.2	96.2	95.5	96.3	92.3	95.2	95.3	94.3
NP	95.9	97.6	95.7	96.1	94.5	94.7	95.4	94.9
SB-AIC	94.1	94.2	95.7	96.9	94.0	91.3	95.4	94.3
SB-BICn	94.6	94.8	95.7	97.0	94.2	88.5	95.4	94.9
SB-HQCn	94.4	94.4	95.7	97.0	94.3	90.7	95.4	94.6

Note: LN and WB refer to parametric log-normal and Weibull models. NP refers to the nonparametric model. SB-AIC, SB-BICn and SB-HQCn refer to SNP-based models using different information criteria. Estimated Monte Carlo standard error of observed coverage $\approx 0.69\%$.

E. Simulation Results for CIF Estimation of Right-censored Data with 3 Competing Risks

Table 4 describes the simulation scenario for 3 competing risks mentioned in the second last parapgraph of Section 6 in the main paper. In this simulation, only independent right censoring with a censoring probability of 35% was considered and the censoring time was generated as for the setting with $J = 2$ described in Section 6 of the main paper. Simulation results are displayed in Tables 5 to 7.

Table 4. Simulation scenario for CIF estimation for 3 competing risks under only right-censoring.

	Mixture representation based scenario				
Scenario	$T \mid D = 1$	$T \mid D = 2$	$T \mid D = 3$	$P_1\%$	$P_2\%$
3 SNP stdnorm 35% RC					
	$SN\,(-0.1, 0.1, \emptyset)$	$SN\left(-0.5, 0.5, \frac{\pi}{9}\right)$	$SN\left(-0.4, 0.5, \frac{\pi}{9}, \pi\right)$	32	28

Note: $T \mid D = j$ is the time to event distribution for event type j conditional on the occurrence of that event type. P_j is the marginal probability of event type j. $SN(\mu, \sigma, \phi)$ is a random variable T satisfying $\log T = \mu + \sigma Z$; where Z has a SNP distribution with a standard normal base density and vector of spherical coordinates ϕ. RC: independent right censoring.

Table 5. Frequency with which the proposed SNP estimators based on AIC, BICn or HQCn, respectively, chose the correct base density and correct polynomial degrees.

		AIC		BICn		HQCn	
Scenario	n	100	500	100	500	100	500
3 SNP stdnorm 35% RC		220	289	196	490	233	425

All frequencies are based on 1000 simulated data sets per scenario.

Table 6. Integrated mean squared error (IMSE) for different estimation methods.

	$n = 100$			$n = 500$		
Scenario	CIF_1	CIF_2	CIF_3	CIF_1	CIF_2	CIF_3
3 SNP stdnorm 35% RC						
LN/SB-HQCn	**0.97** 0.008	**1.66** 0.046	**1.16** 0.015	**1.01** 0.005	**4.38** 0.158	**2.36** 0.063
WB/SB-HQCn	**1.05** 0.017	**1.13** 0.022	**1.08** 0.013	**2.20** 0.066	**2.24** 0.065	**1.85** 0.041
NP/SB-HQCn	**1.22** 0.017	**1.11** 0.013	**1.01** 0.010	**1.24** 0.018	**1.07** 0.017	**0.97** 0.017
SB-AIC $\times 10^4$	2.35	5.67	13.58	0.47	1.10	2.64
SB-BICn $\times 10^4$	2.31	5.64	13.79	0.46	1.15	2.86
SB-HQCn $\times 10^4$	2.35	5.62	13.69	0.46	1.13	2.75

Note: LN/SB-HQCn, WB/SB-HQCn and NP/SB-HQCn are respectively the ratios (in bold) of the AISE of the parametric lognormal, Weibull and the nonparametric models, respectively, versus the SB-HQCn model with corresponding bootstrap standard errors. For each scenario, the last 3 rows give AISE values of SB models based on all information criteria.

Table 7. Observed coverage probabilities of nominal 95% CI for the CIFs at times $0.5t_m$ and t_m.

	$n = 100$						$n = 500$					
Time point	$0.5t_m$			t_m			$0.5t_m$			t_m		
Scenario	CIF_1	CIF_2	CIF_3	CIF_1	CIF_2	CIF_3	CIF_1	CIF_2	CIF_3	CIF_1	CIF_2	CIF_3
3 SNP stdnorm 35% RC												
LN	93.9	87.1	93.3	94.8	95.9	94.7	94.3	62.7	80.0	94.4	90.7	94.7
WB	93.9	91.9	92.4	94.6	94.7	95.3	86.2	88.3	82.6	94.9	95.3	94.4
NP	94.3	96.2	95.5	94.7	94.1	94.9	95.6	95.1	94.6	94.6	95.3	94.3
SB-AIC	93.8	93.3	94.2	94.6	92.4	95.0	94.3	93.9	94.4	94.6	94.0	95.0
SB-BICn	94.2	93.0	93.1	94.6	91.8	95.1	94.0	90.6	92.4	94.7	90.4	94.7
SB-HQCn	93.9	93.4	93.7	94.6	92.1	95.1	94.2	92.5	93.3	94.7	92.8	95.1

Note: LN and WB are the Monte Carlo coverage probabilities of nominal 95% Wald confidence intervals (CIs) based on the parametric 2 x lognormal and 2 x weibull models respectively. SB-AIC, SB-BICn and SB-HQCn are the observed coverage probabilities of different best SNP models. NP is the observed coverage probability of the nonparametric model. The 95% CIs were calculated based on the cloglog transforms of the CIFs. Estimated SE of MC coverage entries $\approx 0.69\%$.

F. Details of Wald-type and Permutation test for the IWD for Comparisons of CIFs

For gathering statistical evidence to reject or not reject the null hypothesis specified in Section 5 of the main paper, a normal distribution as the null distribution is plausible with the variance of the IWD statistic derived using the delta method. Specifically, let the asymptotic covariance matrices for the estimated parameters of the fitted competing risks models for groups 1 and 2 be denoted by, respectively, V_1 and V_2. When $W(\cdot)$ is a deterministic function, the variance of the IWD can be estimated using the delta rule as

$$\sigma^2_{IWD} = \nabla IWD\,(\theta_1, \theta_2)^T \begin{pmatrix} V_1 & 0 \\ 0 & V_2 \end{pmatrix} \nabla IWD\,(\theta_1, \theta_2)$$

where $\nabla IWD\,(\theta_1, \theta_2)$ is the column gradient vector of $IWD\,(\cdot, \cdot)$ as a function of θ_1 and θ_2, the estimates of parameters of the SNP models for groups 1 and 2, respectively. Note that there is no need to obtain information on covariances between parameter estimates from the two SNP models as they are estimated based on independent groups.

Once the IWD statistic and its variance estimate are computed, the p-value can be derived based on comparing the Wald-type statistic $\frac{IWD}{\sigma_{IWD}}$ to the cumulative standard normal distribution. Alternatively, the null distribution and the resulting p-value can be evaluated exactly without relying on a normal approximation by implementing a permutation test or (or Monte-Carlo approximation to it). This is done by rerandomizing the group memembership a large number of times, for each of which the associated IWD is computed and the p-value is the relative frequency of these IWDs observed at least as extreme as the one from the original data set.

G. QQ Plots of P-values and IWD Statistics for the Comparisons of CIFs in IC-2 Scenarios

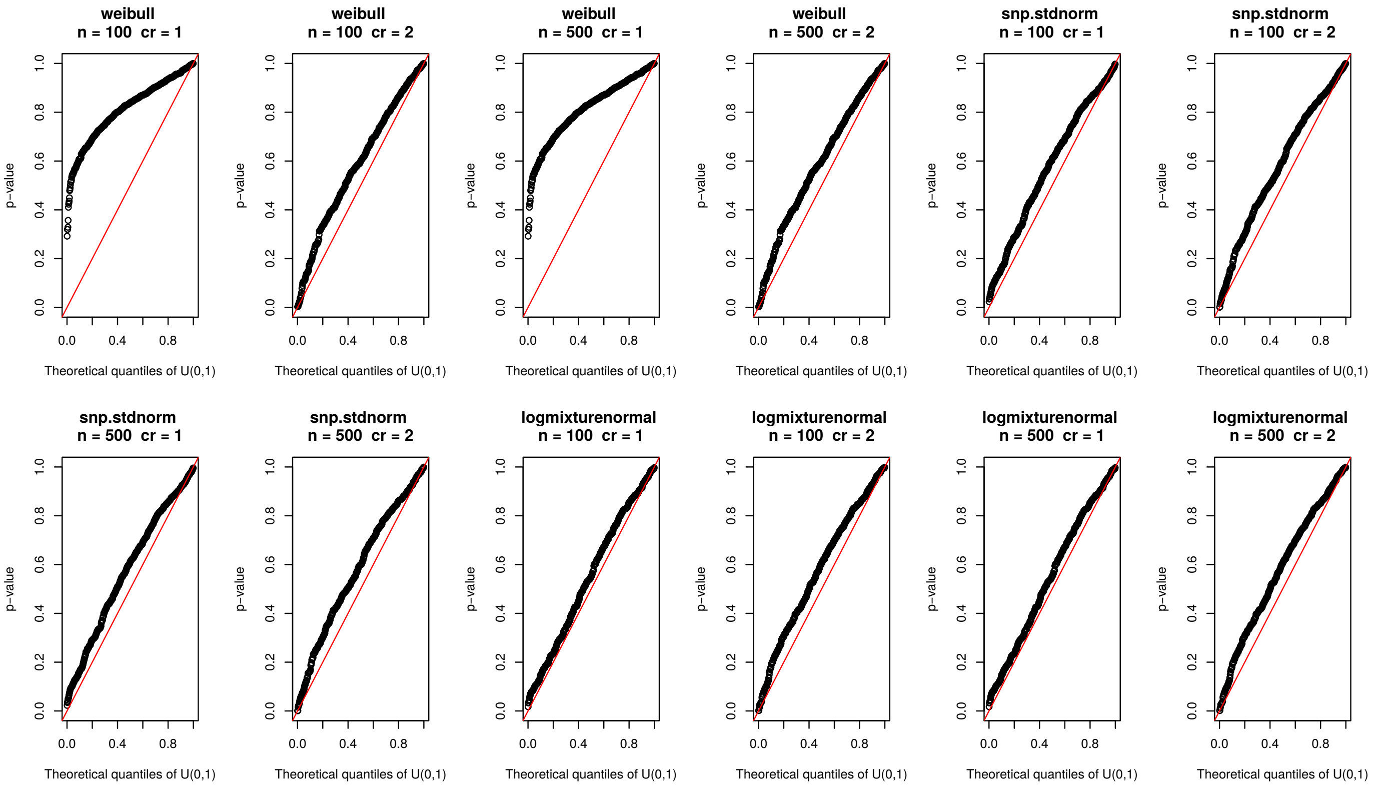

Figure 2. QQ-plots of p-value vs. uniform U(0,1) distribution for IC-2 scenarios.

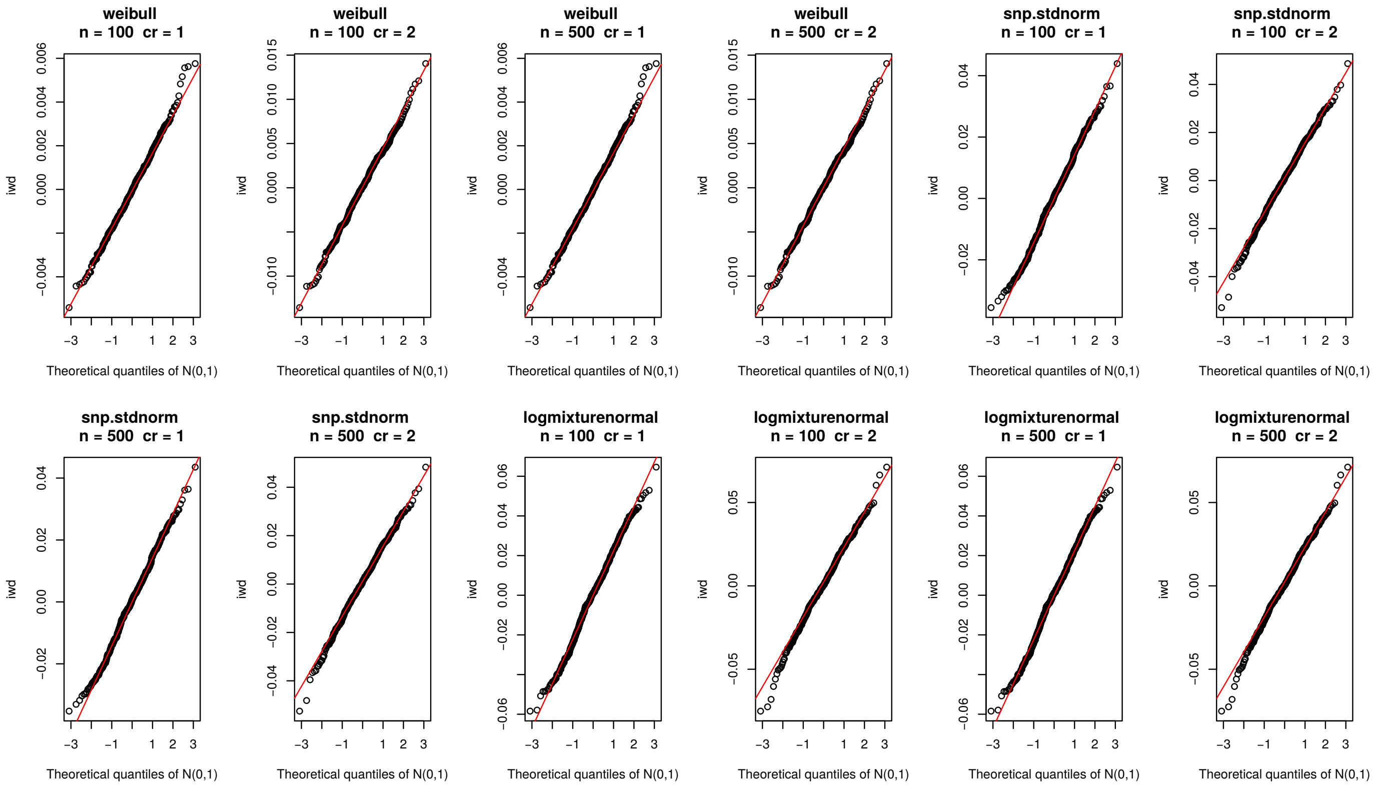

Figure 3. Normal probability plots of IWD statistics for IC-2 scenarios.

References

1. Therneau TM, Grambsch PM. *Modeling Survival Data: Extending the Cox Model*. Springer, New York, 2000.
2. Gray B. cmprsk: Subdistribution Analysis of Competing Risks 2013. URL http://cran.r-project.org/package=cmprsk.
3. Zhang M, Davidian M. "Smooth" semiparametric regression analysis for arbitrarily censored time-to-event data. *Biometrics* 2008; **64**:567–576.
4. Gallant AR, Tauchen G. A nonparametric approach to nonlinear time series analysis: estimation and simulation. *New Directions in Time Series Analysis* 1993; **46**:71–92.
